# Reactions of Dimethylether in Single Crystals of the Silicoaluminophosphate STA-7 Studied via *Operando* Synchrotron Infrared Microspectroscopy

**DOI:** 10.1007/s11244-018-0890-9

**Published:** 2018-01-16

**Authors:** Russell F. Howe, Suwardiyanto, David J. Price, Maria Castro, Paul A. Wright, Alex Greenaway, Mark D. Frogley, Gianfelice Cinque

**Affiliations:** 1 Chemistry Department, University of Aberdeen, Aberdeen, AB24 3UE UK; 2 Chemistry Department, University of Jember, Jember, Jawa Timur 68121 Indonesia; 3 EastCHEM School of Chemistry, University of St Andrews, St Andrews, KY16 9ST UK; 4 EPSRC Catalysis Hub, Research Complex at Harwell, Rutherford Appleton Laboratory, Harwell, OX11 0FA UK; 5 Diamond Light Source, Harwell Science and Innovation Campus, Didcot, OX11 0DE UK

**Keywords:** Infrared microspectroscopy, STA-7, Dimethylether, Hydrocarbon pool

## Abstract

**Abstract:**

Synchrotron infrared micro-spectroscopy has been applied to measure in situ the reaction of dimethylether in single crystals of the silicoaluminophosphate STA-7. The crystals are found to contain a uniform and homogeneous distribution of acidic hydroxyl groups. Dimethylether is hydrogen bonded to the hydroxyl groups at low temperatures, but evidence is found for dissociation to form surface methoxy groups above 473 K, and aromatic hydrocarbon pool species above 573 K. From time resolved infrared measurements coupled with MS analysis of evolved products it is concluded that alkene formation occurs via a direct mechanism from reaction of dimethylether with surface methoxy groups.

**Graphical Abstract:**

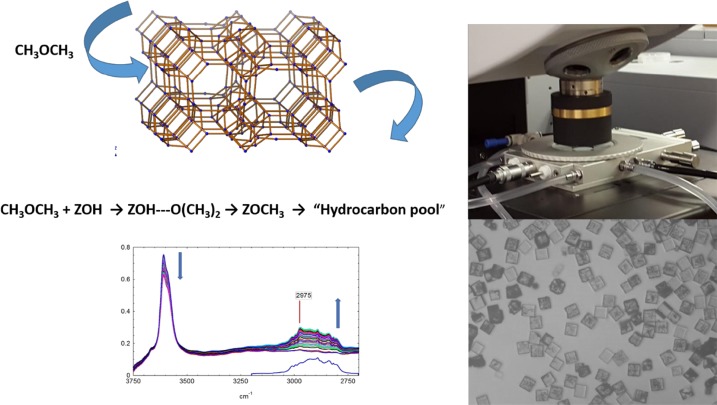

## Introduction

The conversion of methanol to hydrocarbons over acid zeotype catalysts has intrigued the catalysis community ever since Mobil first introduced their MTG (methanol to gasoline) process more than 30 years ago. The original process used an HZSM-5 catalyst to generate a high octane high aromatic gasoline from a methanol–dimethylether stream produced by passing methanol over an alumina dehydration catalyst [1]. Later variations on this technology optimised the formation of olefins at higher temperatures and space velocities over ZSM-5 [2] or utilised smaller pore less acidic SAPO-34 zeotype to selectively produce olefins [3, 4].

From a fundamental viewpoint, the question of how the reaction proceeds and particularly how carbon–carbon bonds are formed from oxygenates such as methanol and dimethylether has attracted wide attention. The recent review by Olsbye et al. summarises the extensive literature on this subject [5]. There is widespread acceptance of the so-called dual cycle hydrocarbon pool mechanism of operation under steady state conditions in which olefinic and aromatic species within the zeolite pores are respectively methylated and cracked to form the observed products. Less well understood are the routes by which the hydrocarbon pool is formed. There is also growing evidence for a so-called direct mechanism for olefin production from methanol during the initial stages of the reaction before the hydrocarbon pool is formed [6–8].

*Operando* spectroscopy has played an important role in identifying species present in the ZSM-5 or SAPO-34 catalysts during methanol conversion. UV–Vis spectroscopy has been used by several groups to identify a range of alkenyl and aromatic carbo cations present in the hydrocarbon pool in both ZSM-5 [9] and SAPO-34 [10]. Similar species are seen by NMR spectroscopy [11]. Stopped flow/quench NMR experiments have revealed species present in the initial stages of the reaction. For example, in ZSM-5 Haw et al. observed a dimethylcyclopentadienyl cation in the initial stages of dimethyl ether conversion which correlated with the first appearance of propene product [12]. In SAPO-34 Dai et al. concluded similarly from NMR that polymethylcyclopentenyl and/or polymethylcyclohexenyl cations formed from alkenes play a key role in the early stages of methanol conversion [13]. Forester and Howe [14] used in situ infrared spectroscopy to show that both methanol and dimethylether formed surface methoxy groups when passed over HZSM-5 at reaction temperatures, and that these methoxy groups could methylate ethene to form propene, and benzene to form toluene. More recently, Yamazaki et al. presented in situ infrared evidence that the methoxy groups in ZSM-5 could form propene by direct reaction with dimethylether [7]. Methoxy groups are also seen by infrared spectroscopy in SAPO-34; Li et al. suggested from *Operando* infrared spectroscopy that the reaction of methoxy groups with dimethylether in SAPO-34 forms a methoxymethyl cation which is the immediate precursor of the initial propene product [8]. Very recently Chowdhury et al. [15] observed adsorbed acetate, methylacetate and dimethoxymethane during the initial stages of methanol conversion over SAPO-34 (using NMR, UV–Vis and MS analysis of evolved products) and proposed a surface species assisted direct mechanism for carbon–carbon bond formation.

Infrared micro-spectroscopy on single crystals of zeotype materials offers several advantages in principle over conventional *Operando* infrared spectroscopy performed on polycrystalline powders either in transmission (as pressed disks) or diffuse reflectance (as loose powders). The homogeneity and uniformity of a catalyst sample can be confirmed by measuring spectra from different crystals or from different spots within one crystal, and orientation of adsorbed species can be studied by polarising the infrared beam relative to the crystal axes. Such measurements can be performed with a conventional laboratory infrared microscope using a spot size down to about 25 μm (defined by setting apertures within the microscope), although the signal to noise levels achieved necessitate long collection times and preclude *operando* studies. This approach has been used for example to observe template species in single crystals of AlPO-5 zeotypes [16].

The two or more orders of magnitude enhancement in brightness of a synchrotron infrared source compared with a laboratory globar source has opened up new opportunities for *operando* spectroscopy on single crystals of zeotype catalysts [17]. Spectra can be measured with aperture sizes down to the diffraction limit of ~ 5 μm, and the enhanced signal to noise means that spectra can be measured much more quickly than is possible with a laboratory instrument. Conventional *operando* FTIR spectroscopy employs transmission through pressed disks of catalyst or diffuse reflectance measurements on loose powders. In such measurements, spectra measured after injection of a pulse of reactant will contain contributions from all of the crystals in the sample, which do not encounter the reactant simultaneously. In micro-spectroscopy, spectra are measured when reactant first encounters one individual crystal. It is this advantage we have found particularly useful in measuring *operando* spectra under reaction conditions. Previous reports of synchrotron infrared micro-spectroscopy applied to zeotype crystals include observation of the oligomerisation of styrene in ZSM-5 [18], the catalytic conversion of 2-chlorothiophene in ZSM-5 [19], the dealumination of individual ZSM-5 crystals [20] and the reactions of methanol and ethanol in single crystals of SAPO-34 [21]. The method has also been applied to study variations in acidity between different fluid catalytic cracking catalyst particles [22].

In this study we report the use of synchrotron infrared micro-spectroscopy to study the reactivity of dimethylether in single crystals of the acidic aluminophosphate zeotype STA-7. STA-7 is a silicon substituted aluminophosphate with eight-ring windows comparable in size to those in SAPO-34. The STA-7 structure contains however two different size cages, one (the A cage) slightly smaller than the cages in SAPO-34, and one (the B cage) slightly larger. STA-7 converts methanol to light olefins with a performance comparable to that of SAPO-34 [23]. Given the suggestion that dimethylether is the immediate precursor of carbon–carbon bond formation in both ZSM-5 and SAPO-34 [7, 8], we chose to study the reactivity of dimethylether in single crystals of STA-7 with the synchrotron infrared micro-spectroscopy technique.

## Materials and Methods

STA-7 crystals were prepared following the procedure described in [22] from a gel with an Al:P:Si ratio of 1.0:0.8:0.2. They were pre-calcined in flowing oxygen up to 823 at 5 K min^− 1^ and held at this temperature for 12 h before being cooled to room temperature. Product phase identification was performed by powder X-ray diffraction on a Stoe STAD diffractometer using monochromatized Cu Kα radiation, and the framework composition determined by energy dispersive EDX analysis in a JEOL JSM-5600 SEM, which was also used to determine crystal sizes. Porosity was measured by N_2_ adsorption at 77 K using a Hiden IGA gravimetric analyser.

The infrared micro-spectroscopy employed a Bruker Hyperion 3000 infrared microscope-fitted with either a ×15 or a ×36 magnification objective & condenser-coupled to a Bruker Vertex 80V FTIR instrument at MIRIAM beam line B22 of Diamond Light Source. Less than 1 mg of precalcined STA-7 crystals were sprinkled onto a CaF_2_ window mounted in a Linkam FTIR600 in situ cell equipped with CaF_2_ windows on the remotely controlled microscope stage. The outer body of the cell was water cooled. The nitrogen gas stream inlet to the cell was controlled by a mass flow controller (typically 100 mL min^− 1^). Reactant dimethylether (research grade, BOC) was injected as a gas flow from a second mass flow controller (typically 20 mL min^− 1^). In the second series of experiments the outlet from the Linkam cell was directed to a Pfeiffer Omnistar quadrupole mass spectrometer with an electron multiplier detector.

Two different experimental protocols were used. The STA-7 crystals were initially dehydrated by heating in flowing nitrogen at 573 K until all traces of adsorbed water were removed from the spectrum, then cooling in flowing nitrogen to the desired reaction temperature. In the first protocol, spectra were collected with the ×36 objective and slits at the sample set to 15 × 15 μm, and the initial temperature set to 423 K. The Bruker OPUS software wizard was used to locate measurement points on typically nine different crystals plus a background measurement off-crystal. Spectra were then recorded from each point with 256 scans and 4 cm^− 1^ resolution, equivalent to 32 s per measurement. A set of spectra was measured in flowing nitrogen at 423 K, then in flowing dimethylether at this temperature, then again in flowing nitrogen after turning off the dimethylether flow. The temperature was then raised to 473 K in flowing nitrogen and the measurements repeated. This sequence was continued with the temperature being progressively raised to 523, 573 and 623 K.

In the second protocol we undertook a series of time resolved isothermal experiments beginning at each temperature with a fresh set of crystals. In this protocol a single background spectrum was measured at the chosen reaction temperature in flowing nitrogen, then the microscope stage moved to locate one individual crystal and the spectrometer set to record repeated spectra comprising 16 scans at 4 cm^− 1^ resolution (2 s per spectrum). These measurements used the 15 × objective and a 20 × 20 µm slit size at the sample. The dimethylether flow was turned on for typically 30 min then off again and spectra recorded continuously during this period. A spectrum of the steady state gas phase dimethylether in the cell was also recorded during this sequence by moving off-crystal. The effluent from the cell was continuously monitored with the mass spectrometer.

## Results and Discussion

### Characterisation of STA-7 Single Crystals

**Fig. 1:**
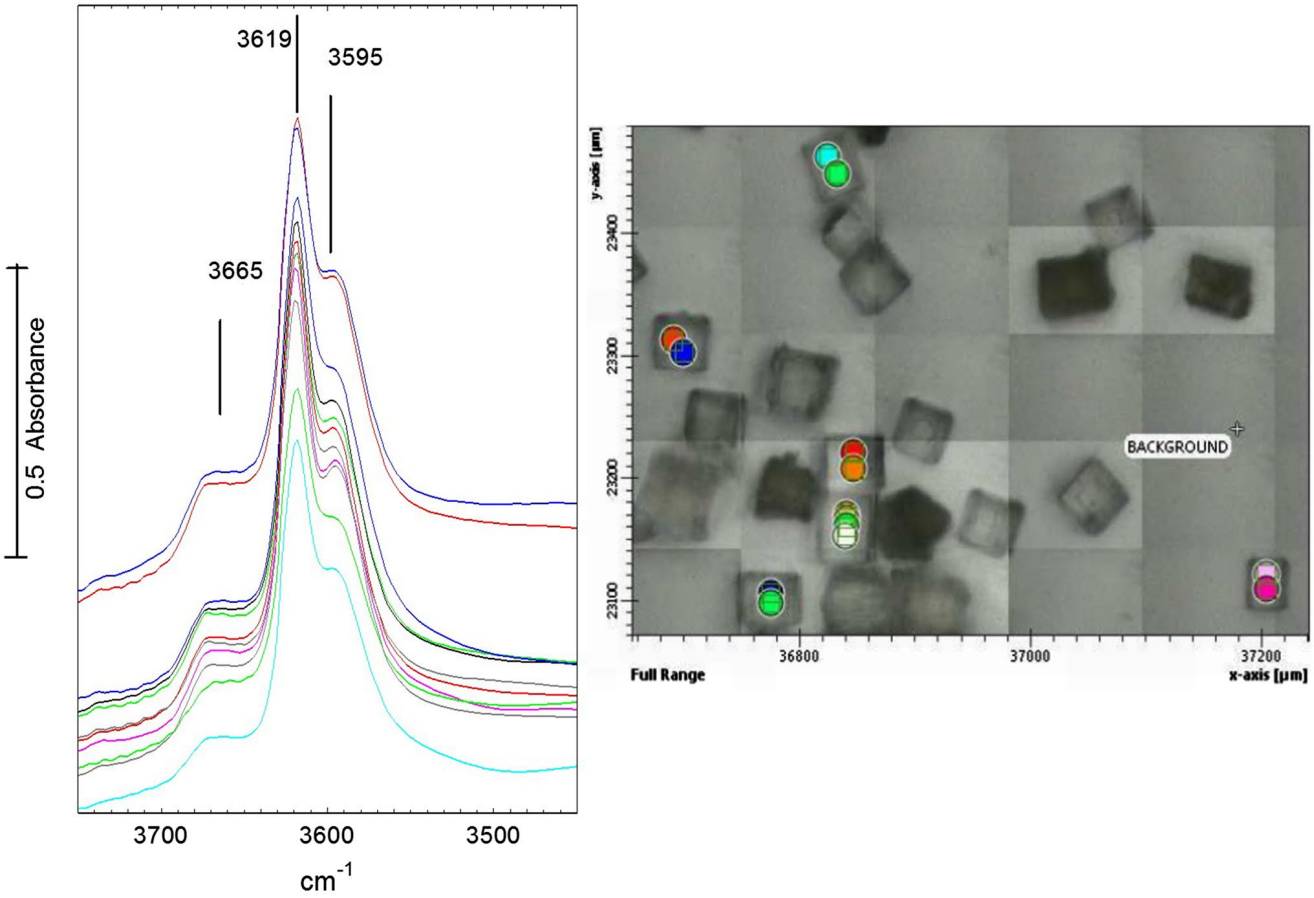
Spectra in the OH stretching region of dehydrated STA-7 crystals, measured at 423 K from the spots marked in the optical image (optical image recorded through the infrared microscope)

STA-7 crystals with a tetrahedral atom compositional ratio Al:P:Si = 1.00:0.73:0.27 by EDX were obtained as tetragonal prisms 40–50 μm in size (see Fig. 1). PXRD indicated the sample was phase pure, as shown previously [23]. Calcination gave a material with a pore volume of 0.29 cm^3^ g^− 1^. Previous solid-state NMR studies on materials prepared similarly [23] showed that the Si substitutes for P in the aluminophosphate lattice.

### Hydroxyl Groups in STA-7

Figure 1 shows infrared spectra measured in flowing nitrogen at 423 K in the OH stretching region from the STA-7 crystals identified in the figure following dehydration at 523 K. Note that the quality of the optical images recorded through the infrared microscope is poor; separate examination in an optical microscope revealed that the crystals were uniformly transparent. Although there are some variations in the overall OH band absorption background due to different crystal thicknesses, the relative intensities of such bands are remarkably consistent across different crystals and from different parts of the same crystal. The two major bands occur at 3619 and 3595 cm^− 1^ with an additional broad higher frequency shoulder at ~ 3665 cm^− 1^. These OH absorption bands are quite similar to those measured by conventional transmission infrared spectroscopy on pressed disks of STA-7 powder by Picone et al. [24]. The two major bands have been assigned to acidic charge balancing protons by comparison with the considerable literature that exists for the chabazite zeolite and SAPO-34 materials with closely analogous structures. In the case of SAPO-34, bands at 3630 and 3600 cm^− 1^ have been assigned to OH groups associated with the O4 and O2 sites in the CHA structure [25]. Martins et al. [26] combined FTIR and ^29^Si NMR to identify a third more acidic OH group at 3617 cm^− 1^ in SAPO-34 attributed to Si sites at the borders of silica islands. Halasz et al. [27] have recently attributed an additional band at 3674 cm^− 1^ which appears very strongly in diffuse reflectance spectra of SAPO-34 and only weakly in transmission spectra to surface AlOH groups. Such surface AlOH groups are not expected to contribute significantly to the transmission spectra of single crystals of STA-7 measured here. The broad higher frequency shoulder at 3670 cm^− 1^ in Fig. 1 is more likely to be due to some partial hydrolysis of the framework during calcination and/or dehydration of the crystals. Regardless, the extent of any such hydrolysis is consistent across all of the crystals measured.

### Reactivity of Dimethylether in STA-7 at Different Temperatures

**Fig. 2:**
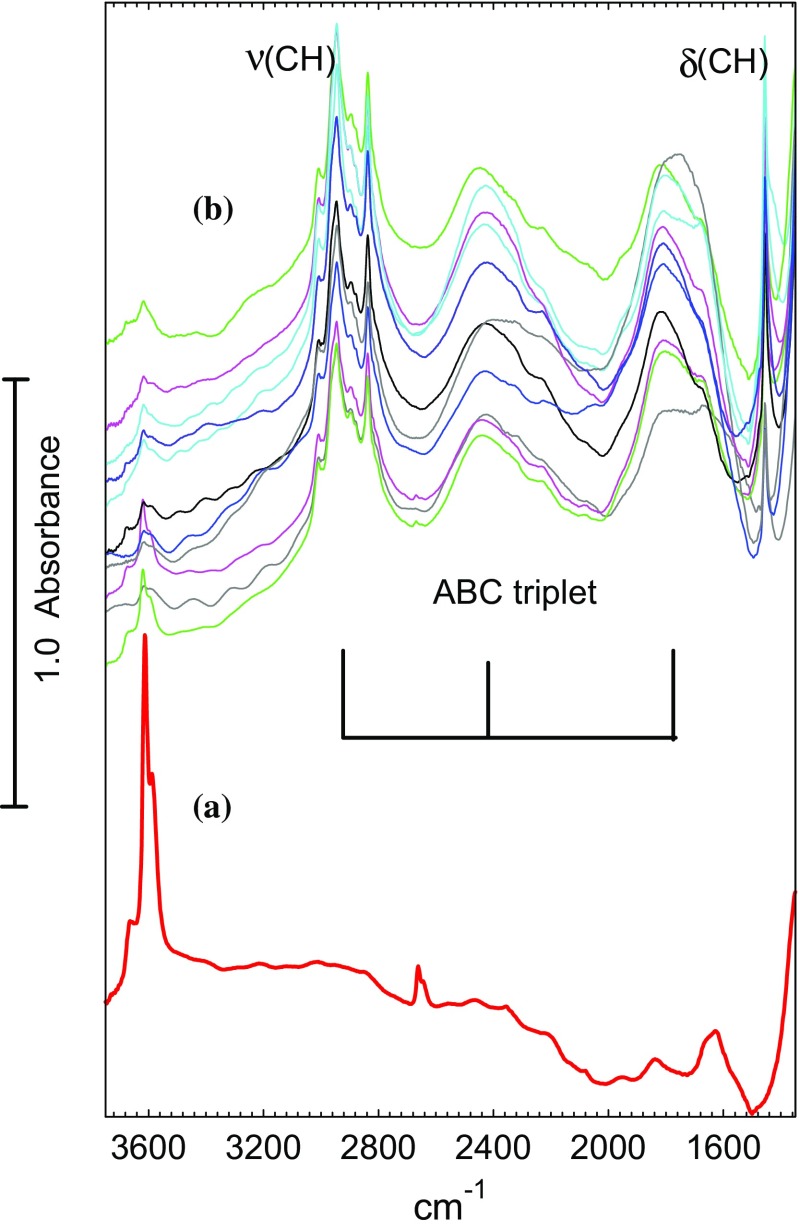
(*a*) Spectrum of a dehydrated STA-7 crystal measured in flowing N_2_ at 423 K (shifted for clarity); (*b*) spectra measured from the crystals marked in Fig. 1 in flowing dimethylether at 423 K

To investigate the interaction of dimethylether with STA-7 crystals at low temperature, dehydrated crystals were held at a temperature of 423 K in flowing nitrogen and a continuous flow of dimethylether was then introduced into the cell and spectra recorded from nine different crystals. A background spectrum was also recorded in the presence of flowing dimethylether so that there are no contributions to the crystal spectra from gas phase dimethylether. Figure 2a shows the spectrum of a typical dehydrated crystal at 423 K prior to admission of dimethylether, and Fig. 2b the set of nine spectra measured from different crystals following admission of dimethylether. No normalisation or subtraction of the spectrum of the unreacted zeolite has been undertaken. The spectra from different crystals are again remarkably consistent, showing only small variations in intensity due to differences in crystal thickness.

In all crystals the intensities of all of the hydroxyl bands are reduced by 90% or more in the presence of dimethylether, indicating that the dimethylether has access to virtually all of the hydroxyl groups within the STA-7 structure at this temperature. The new spectral features appearing are due to hydrogen bonded dimethylether: CH stretching modes at ~ 3000 cm^− 1^, a CH_3_ deformation mode at 1455 cm^− 1^ and a so-called ABC triplet due to hydrogen bonded OH groups at ~ 2900, 2440 and 1760 cm^− 1^. Table 1 summarises the observed frequencies and compares them with related systems in the literature. There have been no previous reports of infrared spectra of dimethylether in STA-7 (or SAPO-34), but comparisons can be drawn with dimethylether in acidic zeolites, particularly ZSM-5.

**Table 1:** Infrared frequencies of hydrogen bonded dimethylether

	Observed frequency (cm^− 1^)	Assignment	Reference
STA-7	2900, 2440, 1760 (all broad)	OH–O(CH_3_)_2_ ABC triplet	This work
	3008(sh), 2960(sh), 2944(s), 2896(w), 2877(w), 2837(s)	ν(CH) and δ(CH_3_) overtones	
	1455(s)	δ(CH_3_)	
HY	2870, 2395, 1780 (all broad)	OH–O(CH_3_)_2_ ABC triplet	[28]
	3010, 2943, 2895,2875, 2830	ν(CH) and δ(CH_3_) overtones	
	1454	δ(CH_3_)	
HZSM-5	2900, 2300, 1700−1500 (all broad)	OH–O(CH_3_)_2_ ABC triplet	[28]
	3010, 2966, 2942, 2829	ν(CH) and δ(CH_3_) overtones	
	1460	δ(CH_3_)	
	~ 3000, ~ 2400, 1700 –1500 (all broad)	OH–O(CH_3_)_2_ ABC triplet	[29]
	3011, 2971, 2947, 2844	ν(CH) and δ(CH_3_) overtones	

Zecchina et al. [28] have reported a detailed analysis of the infrared spectra of various oxygenates hydrogen bonded in acid zeolites HY, HZSM-5 and HMOR. The ABC triplet arises from Fermi resonance between the OH⋯O stretching modes and the overtones of the δ and γ deformation modes of the zeolite hydroxyl groups, producing so-called Evans windows [30] at ~ 2700 and 2000 cm^− 1^ [31]. As discussed by Zecchina et al., the frequencies and relative intensities of the ABC components depend on the strength of the hydrogen bonding and the extent to which the zeolite proton is partially transferred to the oxygen of the adsorbate molecule. The spectra of dimethylether in STA-7 at 423 K (Fig. 2) appear very similar to those reported for dimethylether hydrogen bonded in zeolite HY, suggesting that the acid strength of the protons in STA-7 is similar to that of HY. Note in particular that the three components of the ABC triplet in Fig. 2 have similar intensities, comparable with HY. In contrast, the lowest frequency C component for dimethylether hydrogen bonded in HZSM-5 is shifted to lower frequency and is noticeably more intense than the A and B components [28]. Furthermore, the spectra of dimethylether in HZSM-5 show a pronounced negative feature at 1469 cm^− 1^ superimposed on the C component of the ABC triplet. This additional Evans window results from a direct interaction between the ν(OH⋯O) modes of the hydrogen bonded dimethylether and the δ(CH_3_) modes of the adsorbed dimethylether. This feature is absent from the spectra of dimethylether in STA-7 (and HY). These differences indicate that the acidity of STA-7 is weaker than that of ZSM-5.

Following formation of hydrogen bonded dimethylether at 423 K we subsequently flushed the cell with nitrogen at this temperature, recorded spectra again, then raised the temperature in 50 K steps, recording spectra both on exposure to dimethylether and on subsequent flushing at each successive temperature. Spectra were recorded from the same nine crystals at each temperature, as well as background spectra in nitrogen and in dimethylether. For clarity, we show here the sequence of spectra for one crystal only (the same crystal at each stage), but note that closely similar spectra were measured from all crystals.

**Fig. 3:**
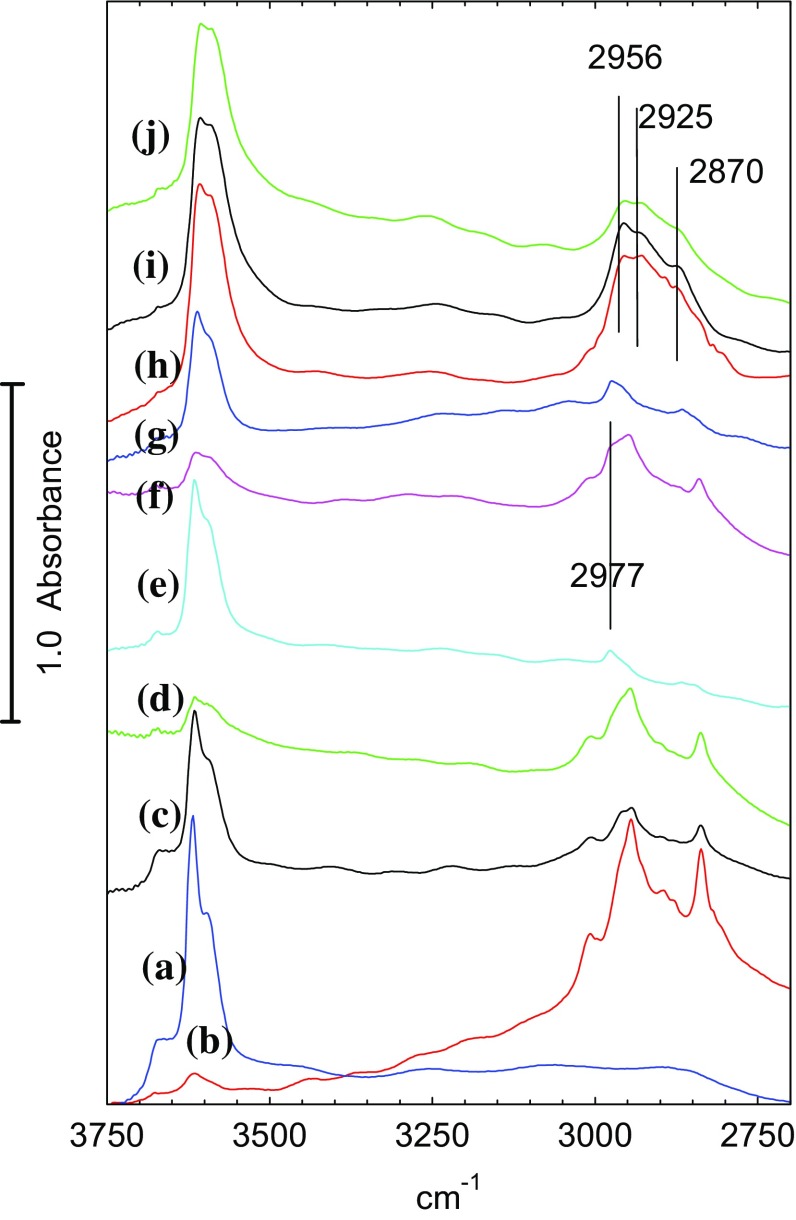
Spectra of (*a*) dehydrated STA7 crystal at 423 K in flowing nitrogen; (*b*) exposed to flowing dimethylether at 423 K for 15 min ; (*c*) subsequently flushed in flowing nitrogen for 20 min; (*d*) exposed to flowing dimethylether at 473 K for 10 min; (*e*) subsequently flushed in flowing nitrogen at 473 K for 10 min; (*f*) exposed to flowing dimethylether at 523 K for 10 min; (*g*) subsequently flushed in flowing nitrogen at 523 K for 10 min; (*h*) exposed to flowing dimethylether at 573 K for 10 min; (*i*) subsequently flushed in flowing nitrogen at 573 K for 10 min; (*j*) exposed to flowing dimethylether at 623 K for 10 min

**Fig. 4:**
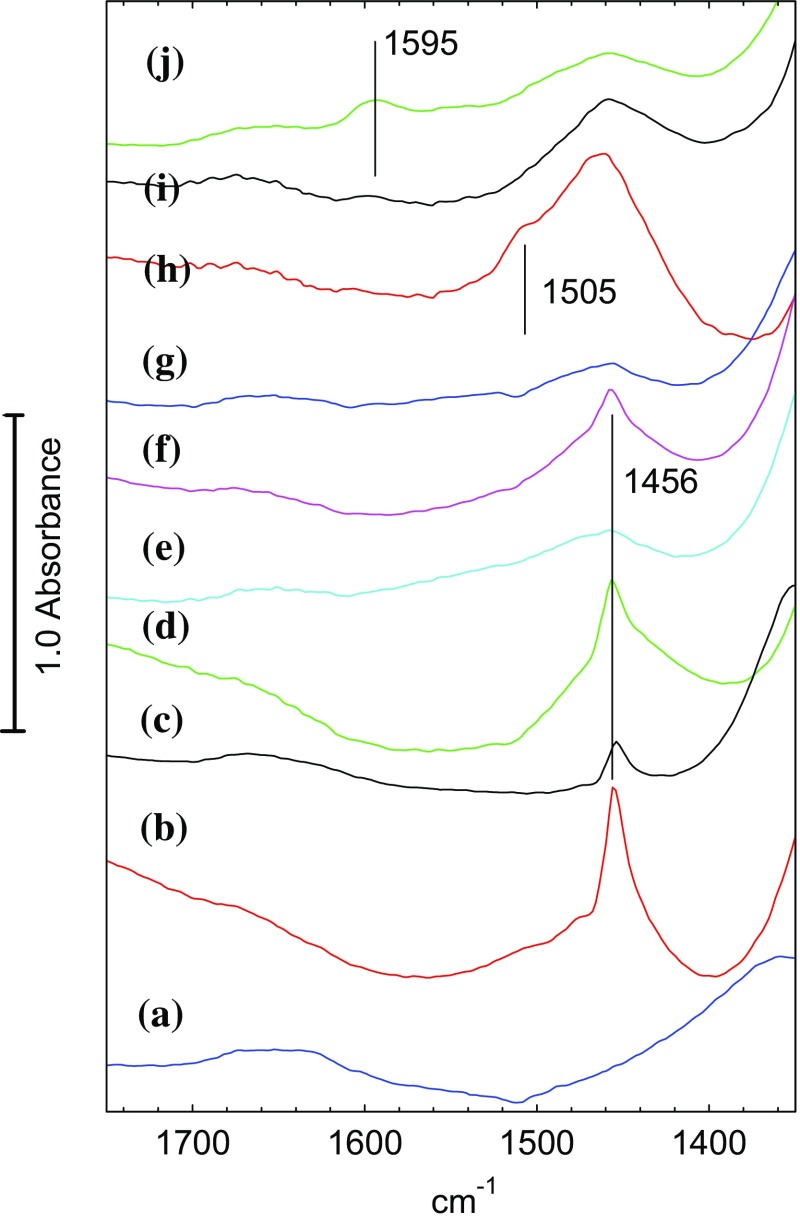
Spectra of (*a*) dehydrated STA7 crystal at 423 K in flowing nitrogen; (*b*) exposed to flowing dimethylether at 423 K for 15 min; (*c*) subsequently flushed in flowing nitrogen for 20 min; (*d*) exposed to flowing dimethylether at 473 K for 10 min; (*e*) subsequently flushed in flowing nitrogen at 473 K for 10 min; (*f*) exposed to flowing dimethylether at 523 K for 10 min; (*g*) subsequently flushed in flowing nitrogen at 523 K for 10 min; (*h*) exposed to flowing dimethylether at 573 K for 10 min; (*i*) subsequently flushed in flowing nitrogen at 573 K for 10 min; (*j*) exposed to flowing dimethylether at 623 K for 10 min

Figure 3 shows spectra recorded in this sequence in the OH and CH stretching region; the corresponding spectra in the deformation region are given in Fig. 4. Comparison of (a) and (b) in each expanded region shows more clearly the formation of hydrogen bonded dimethylether discussed above. Flushing for 20 min in flowing nitrogen at 423 K (Figs. 3c, 4c) removes a large fraction of the hydrogen bonded dimethylether, partly restoring the hydroxyl groups. Subsequent exposure to dimethylether at 473 K restores the bands of hydrogen bonded dimethylether but only to about 50% of the intensity seen at 423 K (Figs. 3d, 4d). At this temperature, the bands of hydrogen bonded dimethylether are completely removed after flushing in nitrogen for 10 min (Figs. 3e, 4e), but residual bands remain in the CH stretching region at 2977 and ~ 2865 cm^− 1^ accompanied by a weak feature in the deformation region around 1460 cm^− 1^. Comparing the OH bands at this stage with those of the freshly dehydrated STA-7 (Fig. 3a) suggests that it is the higher frequency 3619 cm^− 1^ component and the broad 3665 cm^− 1^ shoulder that have been diminished.

The residual CH stretching and deformation bands remaining after flushing at 473 K compare closely with those assigned from previous infrared studies on methanol and dimethylether in ZSM-5 to methoxy groups formed in this case by dissociation of dimethylether:

$${\text{ZOH }}+{\text{ C}}{{\text{H}}_3}{\text{OC}}{{\text{H}}_3} \to {\text{ ZOC}}{{\text{H}}_3}+{\text{ C}}{{\text{H}}_3}{\text{OH}}$$

Table 2 compares the frequencies assigned to surface methoxy groups in ZSM-5 from earlier studies. These experiments with STA-7 indicate that methoxy groups are formed either during adsorption of dimethylether at 473 K or during subsequent desorption (the 2977 cm^− 1^ band cannot be clearly distinguished from the bands due to hydrogen bonded dimethylether during exposure to dimethylether at 473 K).

**Table 2:** Infrared frequencies of methoxy groups in acid zeotypes

	Observed frequency (cm^− 1^)	Assignment	Reference
STA-7	2977, 2865	CH_3_ ν_asymm_ and ν_symm_	This work
	1456	δ(CH_3_)	
SAPO-34	2978	CH_3_ ν_asymm_	[21]
	1456	δ(CH_3_)	
	2977	CH_3_ ν_asymm_	[8]
	2975	CH_3_ ν_asymm_	[10]
	1455	δ(CH_3_)	
ZSM-5	2980, 2871	CH_3_ ν_asymm_ and ν_symm_	[32]
	2978, 2867	CH_3_ ν_asymm_ and ν_symm_	[29]
	2980, 2868	CH_3_ ν_asymm_ and ν_symm_	[14]
	1450	δ(CH_3_)	
	2980, 2868	CH_3_ ν_asymm_ and ν_symm_	[33]
	1457	δ(CH_3_)	

Subsequent further exposure to dimethylether at 523 K restores the bands of hydrogen bonded dimethylether but with the intensity further reduced from that at 473 K (Figs. 3f, 4f), and the 2977 cm^− 1^ band of methoxy groups can now be clearly seen as a shoulder on the side of the dimethylether band at 2944 cm^− 1^. Flushing with nitrogen at this temperature leaves only the bands of methoxy groups at 2977, 2865 and 1460 cm^− 1^ (Figs. 3g, 4g). Comparison of the OH band intensities at this stage with those of the freshly dehydrated crystal at 423 K (Fig. 3a) suggests that about 50% of the hydroxyl groups have been lost due to methoxy group formation, and that, as above, it is the two higher frequency hydroxyl bands that are mostly affected. It should be noted that this comparison assumes the band intensities are independent of temperature, which may not be the case, as there is certainly a temperature dependence in the hydroxyl group frequencies (a shift of ~ 8 cm^− 1^ to lower frequency is observed between 423 and 523 K).

Subsequent exposure to dimethylether at 573 K gives quite different spectra from those seen at lower temperatures (Figs. 3h, 4h). The band profile in the CH stretching region shows little evidence of hydrogen bonded dimethylether, and the 2977 cm^− 1^ band of methoxy groups also cannot be clearly resolved. The ABC triplet of hydrogen bonded dimethylether was no longer seen (this was particularly clear for the B component, which is not overlapped with any other bands).In the deformation region there is a broader band at 1460 cm^− 1^ with a higher frequency shoulder at 1510 cm^− 1^. Of the three OH stretching bands, it is only the two higher frequency components which are reduced. Subsequent flushing at 573 K (Figs. 3i, 4i) leaves the OH bands unchanged, removes the 1510 cm^− 1^ shoulder and reveals the CH stretching band profile to have components at 2956, 2925 and 2870 cm^− 1^. Finally, further exposure to dimethylether at 623 K (Figs. 3j, 4j) leaves the OH stretching bands unchanged, reduces the intensity of the CH stretching and deformation bands, and forms a new band at 1595 cm^− 1^. Flushing at this temperature and exposing subsequently to dimethylether at 650 K caused no further changes in the spectrum i.e. the hydrocarbon species present in the STA-7 pores at this stage are stable.

**Fig. 5:**
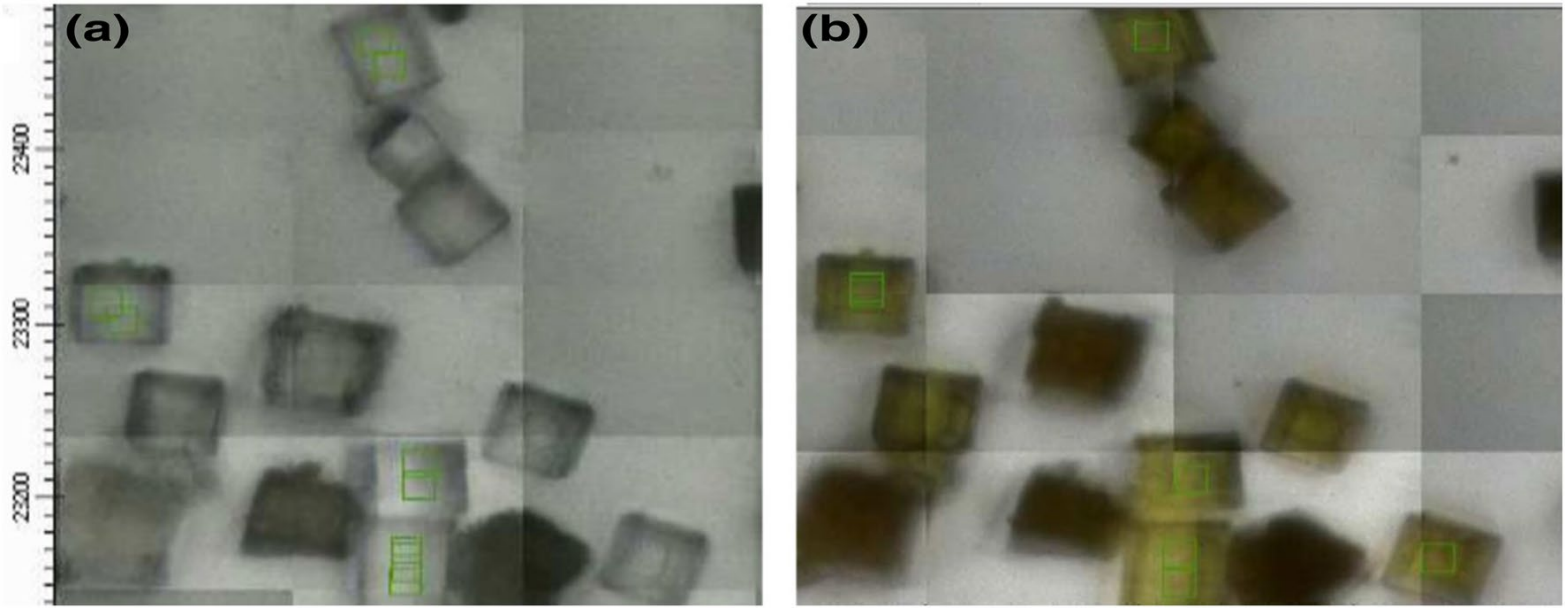
Optical micrographs of **a** freshly dehydrated STA-7 crystals at 423 K; **b** after stepwise heating in dimethylether up to 623 K (images recorded in the infrared microscope)

After this stepwise reaction with dimethylether at temperatures up to 650 K the STA-7 crystals had developed a noticeable yellow colour, as illustrated in Fig. 5. This is consistent with the reported appearance of visible absorption bands in used SAPO-34 MTO catalysts due to polyaromatic species [10]. The apparent variations in colour between crystals are due to thickness effects; note also the comment above about the quality of images recorded through the infrared microscope.

In summary the above experiment has identified the following sequence of events occurring in the STA-7 crystals as the temperature is raised stepwise.

$${\text{C}}{{\text{H}}_3}{\text{OC}}{{\text{H}}_3}+{\text{ ZOH }} \to {\text{ ZOH}} - - - {\text{O}}{\left( {{\text{C}}{{\text{H}}_3}} \right)_2} \to {\text{ ZOC}}{{\text{H}}_3} \to {\text{"Hydrocarbon pool"}}$$

The possible identity of the hydrocarbon pool species formed at and above 573 K and responsible for the new bands appearing in the CH stretching and deformation regions will be discussed further below. Comparison of OH band intensities suggests that almost all of the 3670 cm^− 1^ band and ~ 50% of the 3619 cm^− 1^ OH band have been consumed in forming the hydrocarbon pool, whereas the 3598 cm^− 1^ band is hardly affected. It could be hypothesized that the OH groups responsible for the higher frequency bands are both located in the larger cages of the STA-7 structure, since these will be more accessible to reactant dimethylether. As noted above, similar changes were found in all of the crystals measured in this experiment.

### Time Resolved Measurements with Mass Spectrometric Analysis of Gas Phase Products

To relate the changes in the infrared spectra described above to the catalysis of the dimethylether to hydrocarbon reaction over STA-7, we performed time resolved measurements in which an individual freshly dehydrated STA-7 crystal was held at a particular temperature and spectra recorded at 2 s intervals (16 scans) while the dimethylether flow was turned on and the effluent from the cell analysed continuously with a quadrupole mass spectrometer. In these experiments the infrared spectrometer background is recorded off the crystal prior to turning on the dimethylether flow, so that the spectra recorded when the dimethylether flow is first turned on contain an increasing contribution from the gas phase dimethylether in the cell until a steady state gas phase concentration is achieved. From this point onwards the steady state gas phase spectrum was subtracted leaving spectra of the adsorbed species only.

**Fig. 6:**
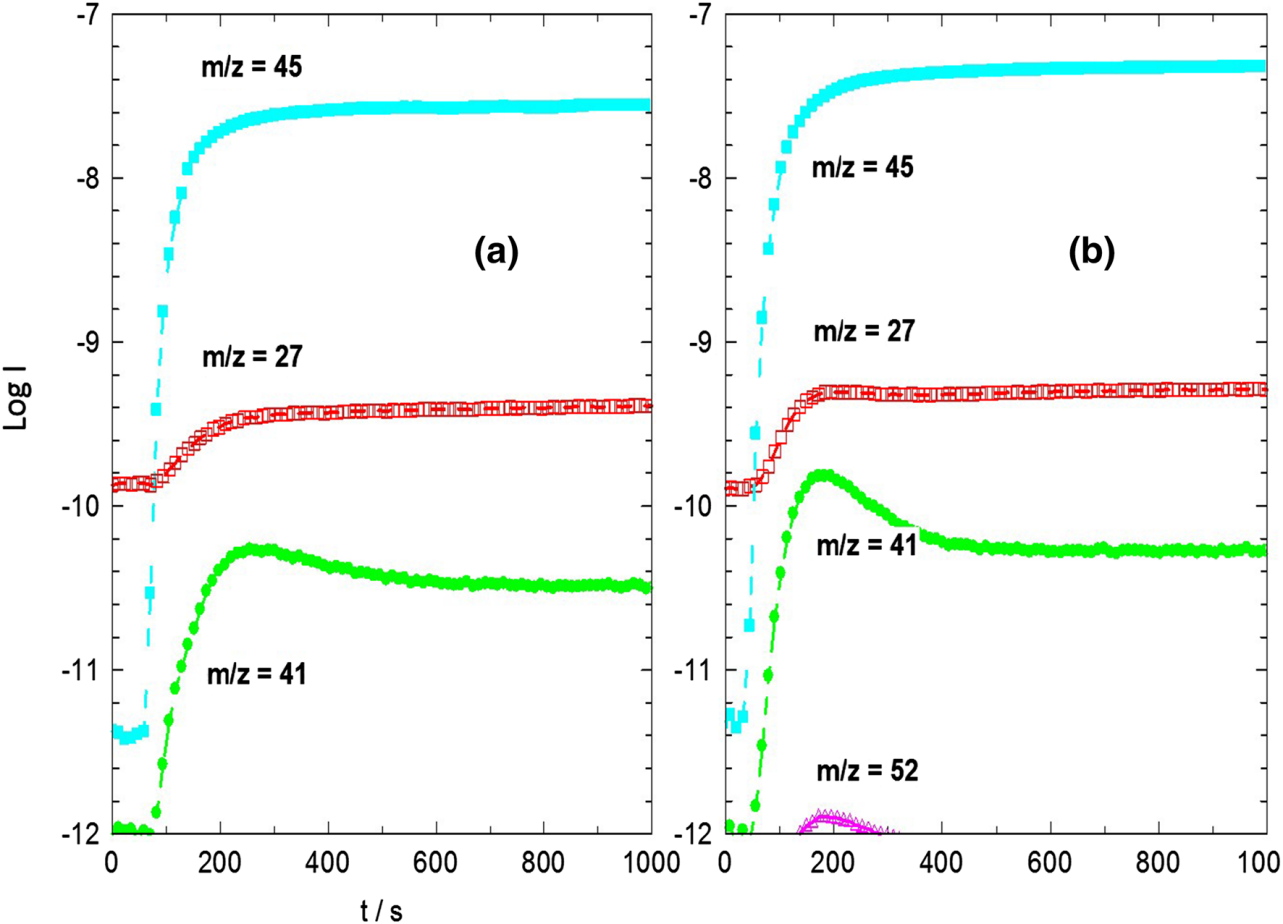
Mass spectral analysis of effluent gas when STA-7 crystals exposed to flowing dimethylether at **a** 623 K; **b** 673 K

Figure 6 shows mass spectrometer traces from two such experiments carried out (with fresh crystals each time) at 623 and 673 K. To maintain good time resolution, the mass spectrometer output was restricted to a small number of masses: m/e = 45 to measure dimethylether, m/e = 41 to measure propene, m/e = 27 to measure ethene and m/e = 56 to measure butene. Fragmentation of the dimethylether in the mass spectrometer does produce contributions at m/e = 41 and m/e = 27. The baseline levels at m/e = 41 and m/e = 27 were confirmed by measuring dimethylether flow through an empty cell containing no STA-7 crystals.

At 623 K there is a small but significant increase in the m/e = 41 signal above the baseline level when the dimethylether flow is first turned on. This declines to the baseline level after ~ 400 s, indicating that propene formation ceases ~ 400 s after first exposure to dimethylether. There is no evidence of ethene formation at this temperature; the m/e = 27 signal follows the baseline expected for dimethylether fragmentation.

At 673 K the propene yield is noticeably higher, but again ceases ~ 400 s after first exposure to dimethylether. At this temperature there is also a small indication of ethene (m/z = 27) and butene (m/z = 56) formation on the same time scale. The mass spectral analysis indicates that at both temperatures the large STA-7 crystals become deactivated for alkene formation after about 400 s on stream. Such deactivation is not unexpected. Stable alkene production from methanol over STA-7 catalysts for more than a few minutes was achieved by Castro et al. only over much smaller crystals [23], and the ~ 10 μm size SAPO-34 crystals studied by Chowdhury et al. [15] deactivated within 10 min.

**Fig. 7:**
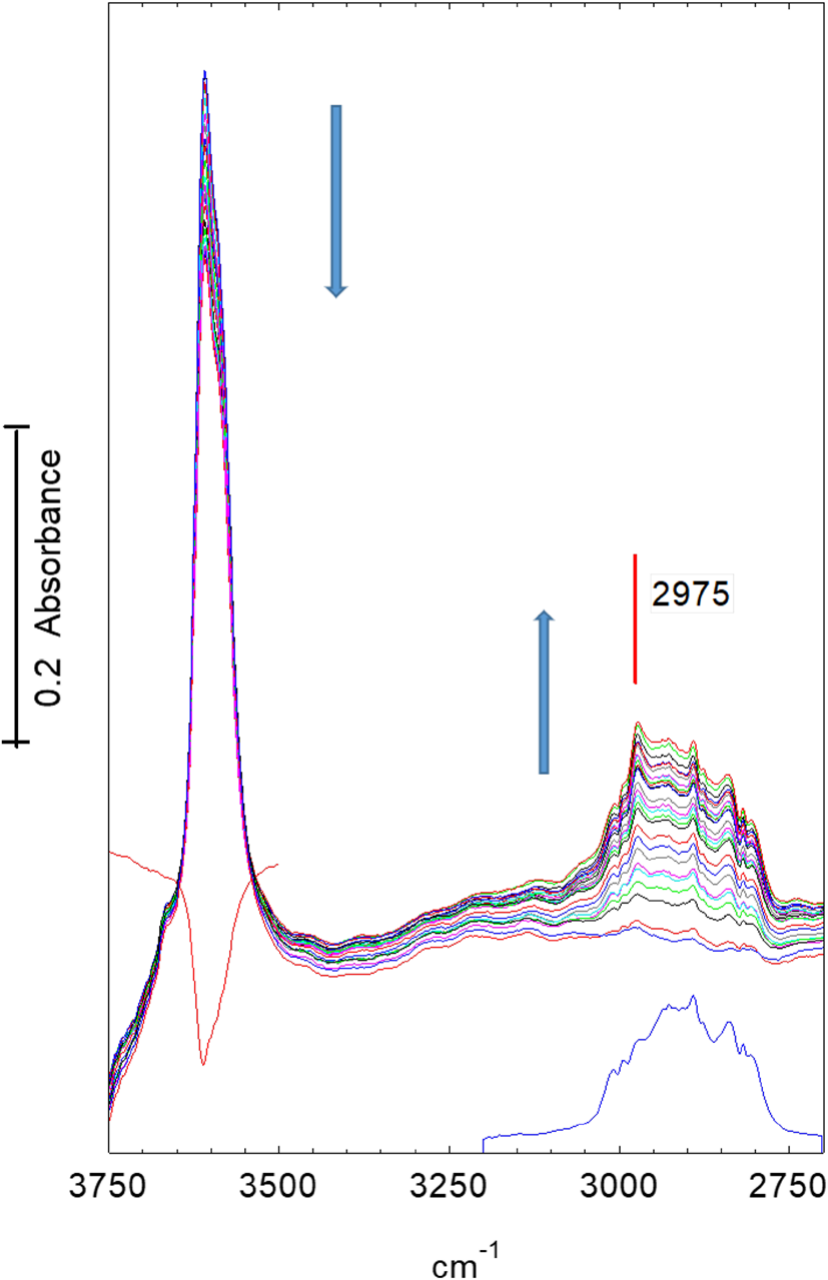
Spectra recorded at 2 s intervals during the first 50 s after exposure of an STA-7 crystal to diemthylether at 623 K. The lower (blue) spectrum is that of gas phase dimethylether in the cell once steady state is achieved (after ~ 50 s). The difference spectrum plotted in red between 3750 and 3500 cm^− 1^ shows the loss of OH band intensity after 50 s. Arrows indicate direction of change

**Fig. 8:**
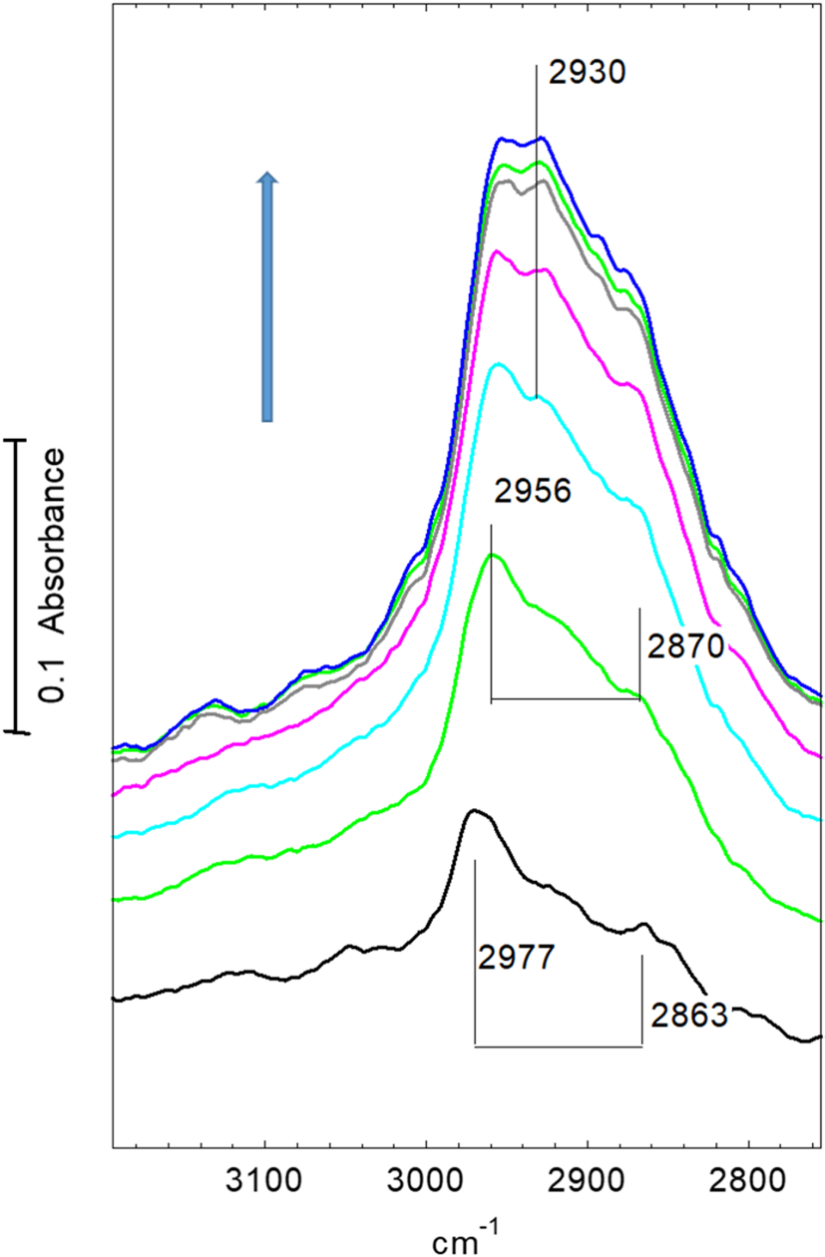
Spectra recorded at 20 s intervals from 80 to 240 s after beginning exposure to flowing dimethylether at 623 K. The gas phase dimethylether spectrum shown in Fig. 7 has been subtracted from each spectrum. The arrow shows the direction of change (spectra have been displaced vertically for clarity)

Figure 7 shows infrared spectra in the OH and CH stretching region recorded at 2 s intervals following introduction of dimethylether into the nitrogen stream at 623 K. There is a gradual reduction in intensity of the OH band at ~ 3609 cm^− 1^ (shifted to lower frequency from 3619 cm^− 1^ at 423 K). In the CH stretching region there is no evidence of hydrogen bonded dimethylether being formed; this was also clearly seen from the absence of the ABC triplet in wider scans. The initial spectra are dominated by bands due to gas phase dimethylether, which reach their steady state intensity after ~ 50 s. There is clearly present however an additional band at 2975 cm^− 1^ due to surface methoxy groups, as described above. The loss of intensity in the 3609 cm^− 1^ OH band corresponds to about 20% of the original intensity, as seen from the difference spectrum shown in the figure. No further changes in the OH region were seen during further exposure to dimethylether for up to 1400 s. The CH band profile did however continue to evolve. Figure 8 shows spectra recorded from 80 to 240 s after exposure to dimethylether. The steady state spectrum of gas phase dimethylether has been subtracted from each of these; for clarity we show every 10th spectrum only. The initial spectrum in Fig. 8 shows clearly the two bands of the methoxy groups described above (2977, 2865 cm^− 1^), but these become replaced in subsequent spectra by the pattern of three bands marked in the figure. These changes in the period 80–100 s after introduction of dimethylether correlate with the observation of propene in the mass spectrum.

**Fig. 9:**
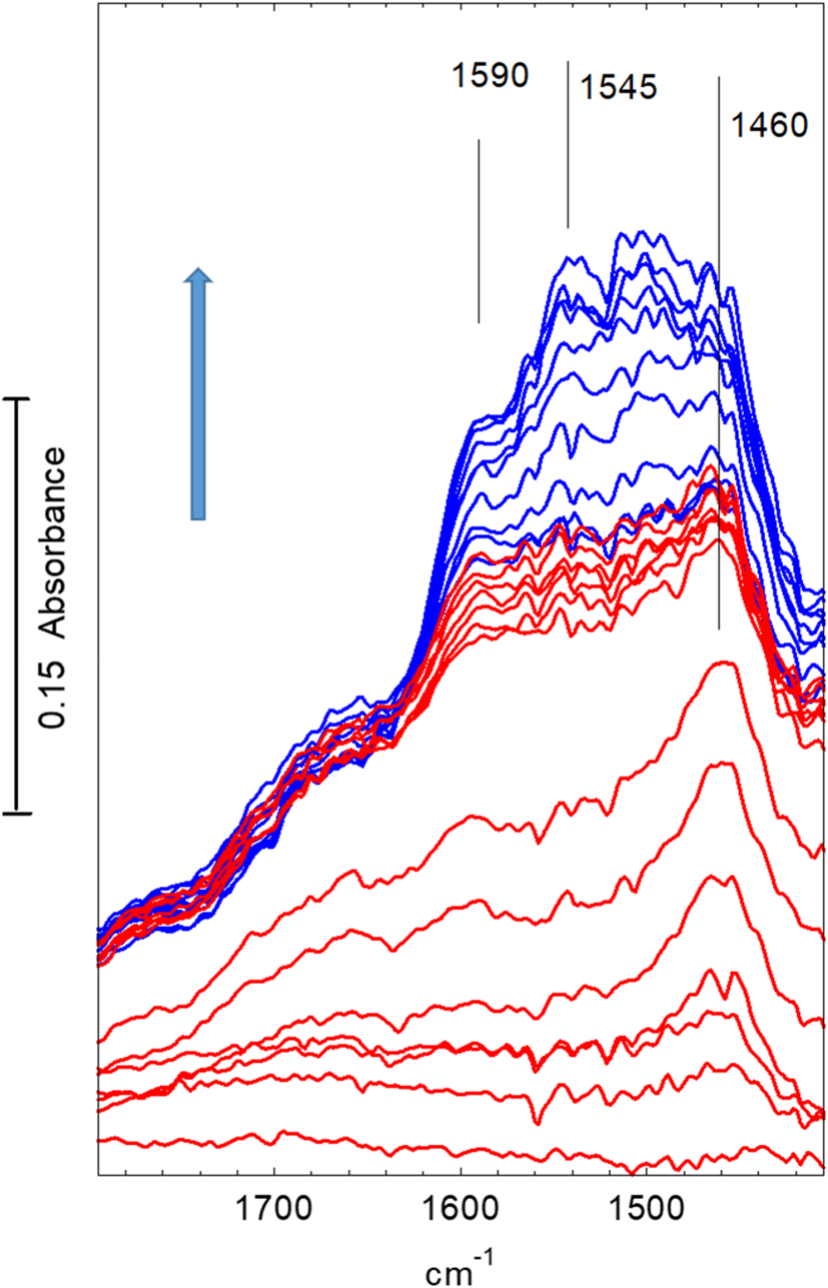
Difference spectra recorded during exposure of STA-7 crystal to flowing dimethylether at 623 K. (The spectrum of the dehydrated crystal at 623 K has been subtracted from each spectrum.) Spectra shown at 20 s intervals for the first 280 s (red traces) then at 100 s thereafter (blue traces). Arrow indicates direction of change. Spectra displaced vertically for clarity

Corresponding changes in the deformation region—are shown in Fig. 9. Every 10th spectrum is shown for the first 280 s (red traces) and every 50th spectrum thereafter (blue traces). The background spectrum of the dehydrated crystal has been subtracted from each spectrum for clarity (the gas phase dimethylether bands in this region are much weaker and have not been subtracted.) A weak band at ~ 1460 cm^− 1^ appears in the first 20–40 s. At a later stage this is overtaken by series of poorly resolved bands between 1595 and ~ 1460 cm^− 1^. Note that these bands continue to grow long after propene formation has ceased.

**Fig. 10:**
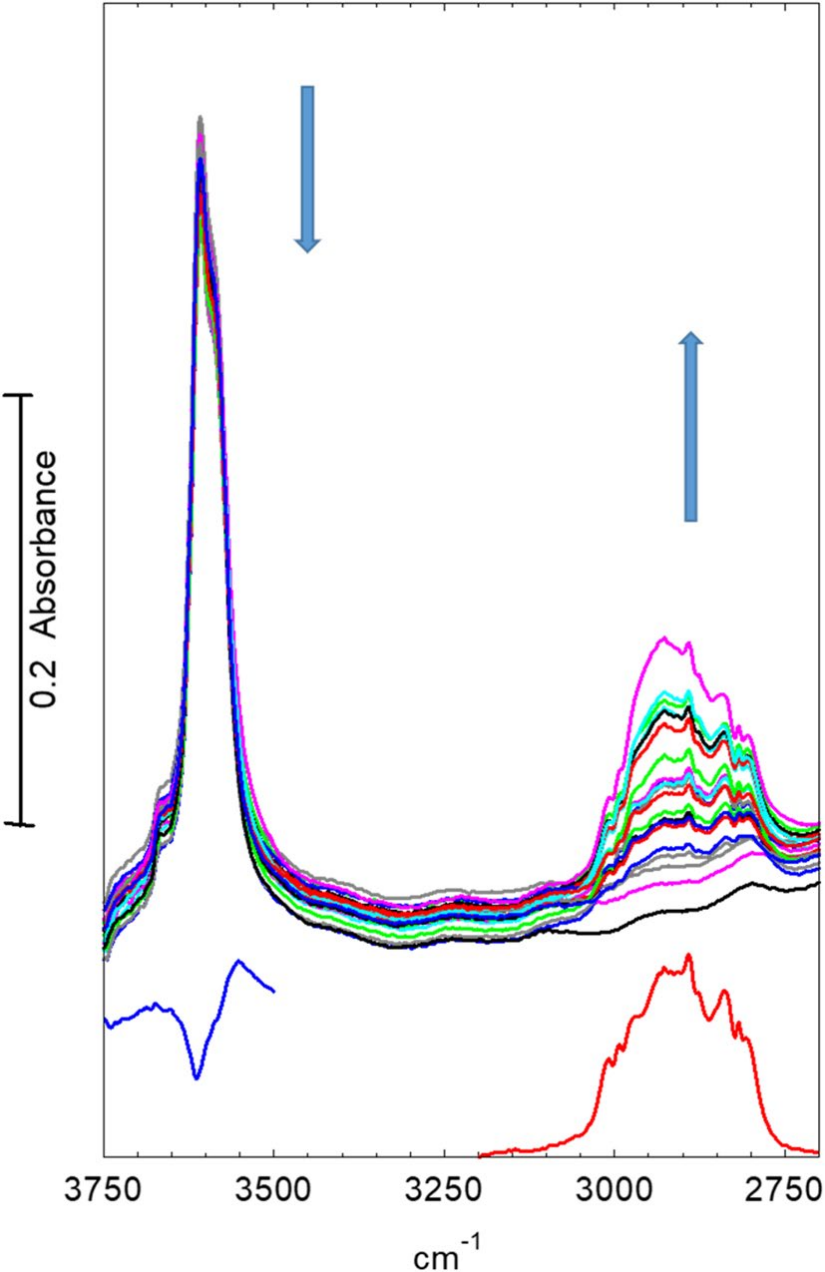
Spectra recorded at 2 s intervals during the first 50 s of exposure of an STA-7 crystal to flowing dimethylether at 673 K. The lower spectrum (red) is that of gas phase dimethylether in the cell once steady state flow is achieved (after ~ 50 s). Also shown (blue) is a subtracted spectrum showing the loss of hydroxyl band intensity after 50 s. Arrows indicates directions of change

**Fig. 11:**
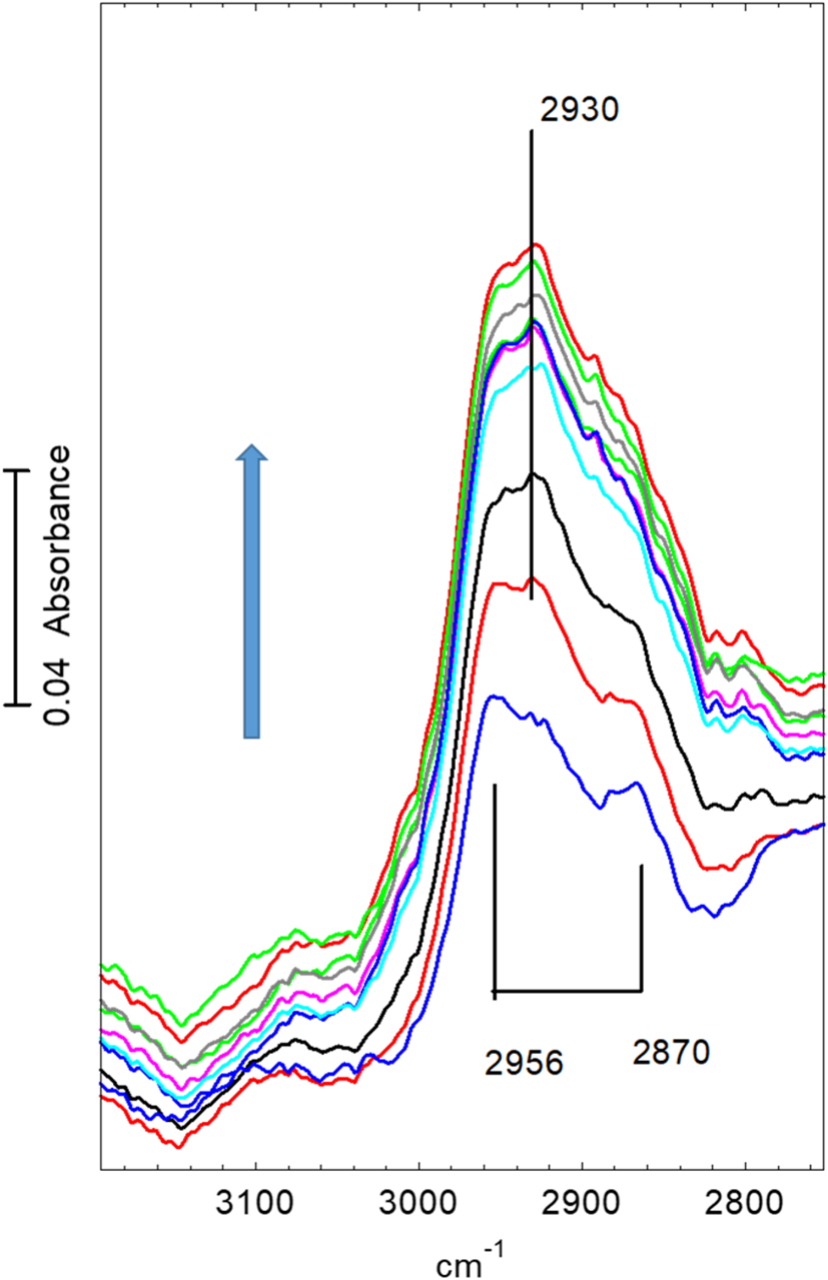
Spectra recorded at 20 s intervals from 60 to 280 s after beginning exposure of STA-7 crystal to flowing dimethylether at 673 K. The gas phase dimethylether spectrum shown in Fig. 10 has been subtracted from each spectrum. Arrow indicates the direction of change

A similar experiment was undertaken at 673 K. Fig. 10 shows infrared spectra in the OH and CH stretching region recorded at 2 s intervals following introduction of dimethylether into the nitrogen stream at 673 K. At this temperature, the loss in intensity in the OH region following introduction of dimethylether is much less than at 623 K (~ 10% reduction, as seen from the subtracted spectrum in the OH region). In the CH stretching region the only obvious bands appearing during the first 50 s are those due to gas phase dimethylether. After longer exposures and subtraction of the steady state gas phase spectrum (Fig. 11) a similar band profile appears to that seen at 623 K, with the notable differences that the bands of methoxy groups are absent, and the other new bands are already present within 60 s of introducing dimethylether.

**Fig. 12:**
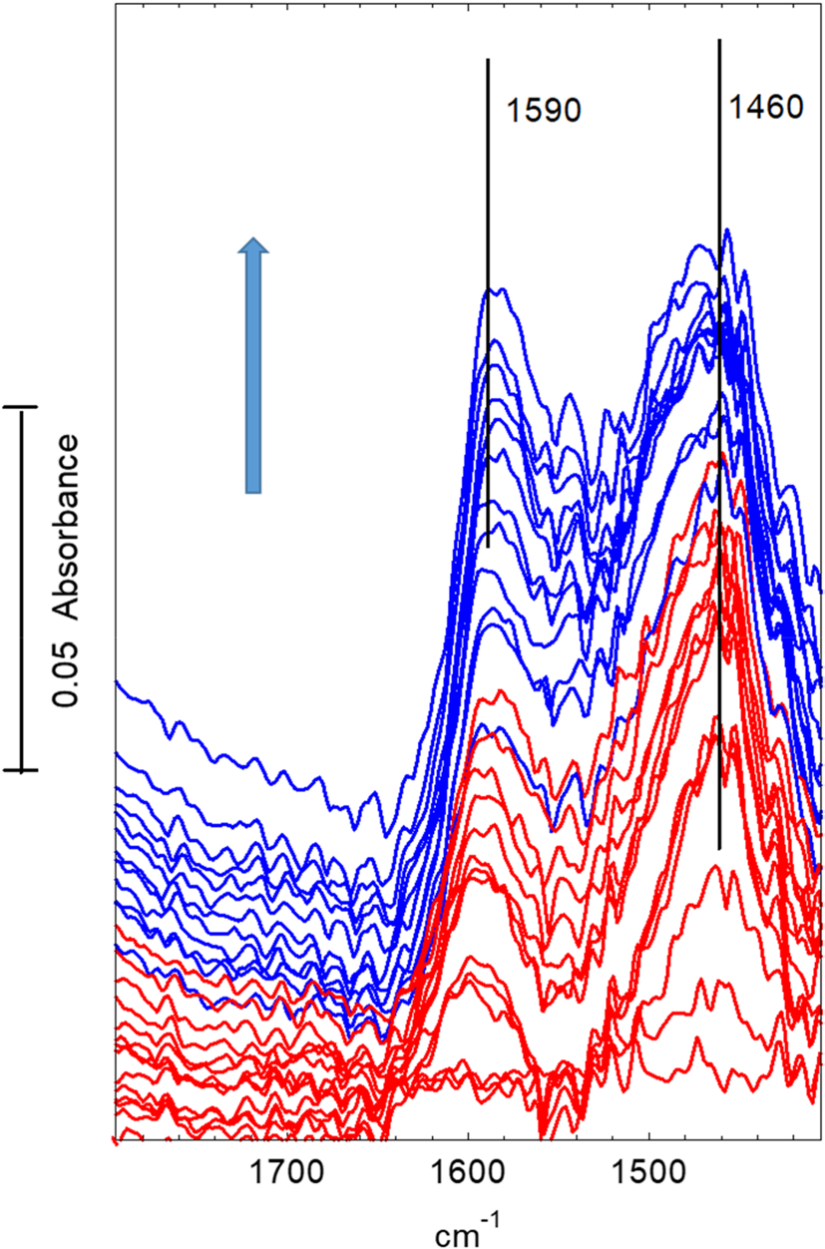
Difference spectra recorded during exposure of an STA-7 crystal to flowing dimethylether at 673 K. (The spectrum of the dehydrated crystal at 673 K has been subtracted from each spectrum.) Spectra shown at 20 s intervals for the first 280 s (red traces) then at 100 s thereafter (blue traces). Arrow indicates direction of change. Spectra displaced vertically for clarity

Corresponding changes in the deformation region are shown in Fig. 12. (The spectrum of the dehydrated crystal has been subtracted in each case.) At 673 K the new bands which evolve in this region grow steadily with time, but do not reach their final intensities until after about 1000 s of exposure.

In the following section we discuss possible assignments for the new infrared bands in the context of previous published studies on SAPO-34. These time-resolved measurements are first of all confirming that the first step in dimethylether conversion is the formation of surface methoxy groups; these are clearly seen as an intermediate species at 623 K, whereas at 673 K their lifetime is too short for them to be seen. The formation of alkene products appears to correlate with the conversion of the methoxy groups and the growth of the new species discussed below.

### Nature of the Hydrocarbon Species in STA-7

In the STA-7 single crystals studied here, the spectra obtained after exposure to dimethylether for extended periods at high temperature are completely consistent with the presence of species observed by UV–Vis [10, 11, 34, 35], NMR [6, 11, 34] and FTIR [8, 10, 21] spectroscopies in SAPO-34. In particular, the CH stretching band profile measured at 573 K or above is very similar to that reported in Qian et al. synchrotron infrared microspectroscopy study of methanol and ethanol conversion in 50 μm crystals of SAPO-34 [21]. Methyl-substituted aromatic species (e.g. tetra, penta and hexamethylbenzenes) give a characteristic pattern of CH_3_ stretching bands centred on ~ 2930–2940 cm^− 1^, deformation modes between 1440 and 1470 cm^− 1^, and C–C stretching modes between 1500 and 1600 cm^− 1^. The spectra measured in the sequential heating experiments show such bands appearing following exposure to dimethylether at 573 K for 10 min (Figs. 3, 4h). Also appearing (and becoming more evident at 623 K) is a band at 1595 cm^− 1^ which may be due to highly conjugated polyaromatic species [10].

It is noteworthy in the sequential heating experiments that although almost all of the acidic hydroxyl groups are accessible to dimethylether at temperatures up to 523 K, above this temperature only a fraction of the original hydroxyl band intensity is lost. This can be explained by reactions occurring in the outer regions of the crystal to form hydrocarbon pool species (e.g. methylated aromatics) which block the pores and prevent any further access of dimethylether to internal sites. Formation of “coke” in the outer regions of large SAPO-34 crystals has been seen by confocal fluorescence microspectroscopy [35], as well as the infrared microspectroscopy in reference [21].

The time resolved experiments allow the sequence of events occurring in the crystals to be followed more clearly. Dissociation of dimethylether to form surface methoxy groups precedes the first observation of alkene products in the gas phase. The sequential heating experiments (Fig. 3e) show that methoxy group formation occurs already at 473 K; thus the reaction of methoxy groups with dimethylether at higher temperatures is clearly the key step in initiating hydrocarbon formation. This conclusion is completely consistent with the so-called direct mechanism for hydrocarbon formation proposed for methanol conversion to hydrocarbons over both ZSM-and SAPO-34 [6–8].

In the time resolved experiments at 623 K (Figs. 7, 8) methoxy groups are detected as a short lived (< 100 s) intermediate preceding the appearance of more intense bands in the CH stretching region. At 673 K (Figs. 10, 11) this species is not detected at all, presumably because its lifetime is too short. Closer examination of the CH stretching region at both temperatures reveals a profile of three overlapping bands (as marked in the figures) which do not maintain fixed relative intensities: bands at 2956 and 2870 cm^− 1^ appear first and are overtaken by a band at 2930 cm^− 1^. These bands appear more quickly at 673 K than at 623 K although the overall intensity of the CH stretching bands is noticeably less at the higher temperature. Faster formation of hydrocarbon pool species at 673 K causes more pore blocking at the edges of the crystals and further reduces the availability of acid sites in the interior of the crystal, so that the overall amounts of adsorbed hydrocarbon species are less than at 623 K. In the deformation region a band at 1460 cm^− 1^ attributed to CH_3_ deformation modes appears at an early stage whereas the 1590 and 1545 cm^− 1^ bands continue to grow during the full duration of exposure to dimethylether. It is particularly noteworthy that these bands continue to grow in intensity after propene formation (at 623 K) or ethene, propene and butene formation (at 673 K) have ceased. We suggest that these bands are due to the aromatic and/or polyenyl hydrocarbon pool species detected in SAPO-34 by UV–Vis and NMR spectroscopies.

It is the appearance of the 2956 and 2870 cm^− 1^ bands in the CH stretching region (along with the 1460 cm^− 1^ CH_3_ deformation band) which correlate best with the appearance of olefin gas phase products in the time resolved experiments. Although we cannot at this stage identify with certainty the species responsible for these bands, some discussion of what this species may or may not be is warranted. Direct methylation of the oxygen in dimethylether by a surface methoxy group would form the trimethyloxonium cation (TMO^+^). As discussed by others [5] this is one potential route to carbon–carbon bond formation via an oxonium ylide, although theoretical calculations suggest this route is unfavourable. The vibrational spectrum of TMO^+^ has been reported (as the SbCl_6_^−^ salt) [36] and is characterised by intense CH stretching modes at 3073, 3052 and 2968 cm^− 1^. Such bands are not seen in the spectra reported here. Alternatively, in the model suggested by Li et al. [8] surface methoxy groups in SAPO-34 react directly with dimethylether to form the methoxymethyl cation, which then reacts with a further dimethylether to form 1,2 dimethoxyethane or 2 methoxyethanol, both of which contain carbon–carbon bonds. The infrared evidence for this model was a band at 2960 cm^− 1^ assigned to the asymmetric CH_2_ stretching vibration of surface bound CH_3_OCH_2_ groups.

In the infrared experiments of Celik et al. cited by Li et al. [37] the surface bound CH_3_OCH_2_ groups formed by dissociate chemisorption of dimethoxymethane (CH_3_OCH_2_OCH_3_) at the acid sites in zeolite HY show multiple bands in the CH stretching region due to symmetric and asymmetric stretching of both CH_3_ and CH_2_ groups (the most intense of these being at 2962, 2912 and 2858 cm^− 1^). These do not match closely the band seen here in STA-7 at 2956 and 2870 cm^− 1^, although Celik et al. note that other species may also be present in their spectra. Adsorbed dimethoxymethane has been seen in SAPO-34 in the recent NMR experiments of Chowdhury et al. [15]. One feature of the spectra of adsorbed dimethoxymethane and related oxygen containing species is the low intensity of the CH_3_ and CH_2_ deformation bands relative to the CH stretching modes (~ 20%), which is also the case in STA-7. What we do not see in the STA-7 spectra are bands in the carbonyl stretching region (1600–1800 cm^− 1^) which would be expected if monodentate formate or acetate species are present. For example chemisorption of methyl formate in zeolite HY gives a C=O stretching band at 1734 cm^− 1^ [37] assigned to a surface formate species. Thus in STA-7 under the conditions of these experiments we see no evidence for the surface bound formate/acetate species C–C bond formation route proposed for SAPO-34 [15, 38, 39].

Notwithstanding our inability to uniquely identify the species initially formed by reaction of dimethylether with surface methoxy groups, we can represent the processes occurring in the STA-7 crystals under reaction conditions by the following scheme (which is consistent with results obtained from both experimental protocols employed):

$${\text{SiOC}}{{\text{H}}_3}{\text{Al }}+{\text{ C}}{{\text{H}}_3}{\text{OC}}{{\text{H}}_3} \to {\text{``C}}{{\text{H}}_3}{\text{OC}}{{\text{H}}_2}\,{\text{ads''}}{\text{ or similar }} \to {\text{ }}{{\text{C}}_3}{{\text{H}}_6} \to {\text{``aromatic hydrocarbon pool''}}$$

The time-resolved experiments have shown clearly that propene (and probably ethene and butene) formation occurs via a direct mechanism. The hydrocarbon pool species, which appear to be mostly aromatic from the infrared spectra, continue to evolve with time after olefin formation has ceased. In these large crystals the blockage of pores near the external surface by hydrocarbon pool species poisons olefin production and a steady state hydrocarbon pool (indirect) mechanism which may well operate in smaller crystals is not seen here.

## Conclusions

The brightness of the synchrotron infrared microbeam has allowed us to perform *Operando* infrared spectroscopy on a 2 s time scale and a 10–20 μm spatial scale. These experiments have provided insight for the first time into how the formation of olefins from dimethylether occurs in STA-7. The method has demonstrated the uniformity of STA-7 crystals and of the adsorbed species generated from dimethylether (at least from the limited data set of six different crystals). Further beam time is needed for a full statistical analysis over many more crystals. The time resolution achieved, coupled with mass spectrometric analysis of evolved gases, has allowed *operando* measurements to reveal the reaction sequence leading to olefin formation and the slower evolution of the hydrocarbon pool within single crystals of STA-7. Many features of the chemistry show strong similarity with what has been observed via other techniques in the closely related SAPO-34 catalyst. The formation of methoxy groups from dimethylether precedes the first observation of propene product. The first appearance of propene correlates with the appearance of infrared bands which may be due to dimethoxymethane formed from methylation of dimethylether, although that assignment remains uncertain. We do not see the formate/acetate species identified in SAPO-34 [15, 38, 39], and further work is needed to understand these differences.

## References

[1] Maiden CJ (1988). Stud Surf Sci Catal.

[2] Koempel H, Liebner W (2007) In: Proceedings of the 8th Natural Gas Conversion Symposium, Natal, Brazil, pp 261–281

[3] Chen JQ, Bozzano A, Globver B, Fuglerud T, Kvisle S (2005). Catal Today.

[4] Tian P, Wei Y, Ye M, Liu Z (2015). ACS Catal.

[5] Olsbye U, Svelle S, Lillerrud KP, Wei ZH, Chen YY, Li JF, Wang JG, Fan WB (2015). Chem Soc Rev.

[6] Wang W, Buchholz A, Seiler M, Hunger M (2003). J Am Chem Soc.

[7] Yamazaki H, Shima H, Imai H, Yokoi T, Tatsumi T, Kondo JN (2012). J Phys Chem C.

[8] Li J, Wei Z, Chen Y, Jing B, He Y, Dong M, Jiao H, Li X, Qin Z, Wang J, Fan W (2014). J Catal.

[9] Mores D, Kornatowski J, Olsbye U, Weckhuysen BM (2011). Chem Eur J.

[10] Qian Q, Vogt C, Mokhtar M, Asiri AM, Al-Thabaiti SA, Basahel SN, Ruiz-Martinez J, Weckhuysen BM (2014). ChemCatChem.

[11] Wang W, Jiang Y, Hunger M (2006). Catal Today.

[12] Haw JF, Nicholas JB, Song W, Deng F, Wang Z, Xu T, Heneghan CS (2000). J Am Chem Soc.

[13] Dai W, Wang C, Dyballa M, Wu G, Guan N, Li L, Xie Z, Hunger M (2014). ACS Catal.

[14] Forester TR, Howe RF (1987). J Am Chem Soc.

[15] Chowdhury AD, Houben K, Whiting GT, Mokhtar M, Asiri AM, Al-Thabaiti SA, Basahel SN, Baldus M, Weckhuysen BM (2016). Angew Chem Int Ed.

[16] Popescu SC, Thomson S, Howe RF (2001). Phys Chem Chem Phys.

[17] Stavitski E, Weckhuysen BM (2010). Chem Soc Rev.

[18] Stavitski E, Kox MHF, Swart I, de Groot FMF, Weckhuysen BM (2008). Angew Chem Int Ed.

[19] Kox MHF, Domke KF, Day JPR, Rago G, Stavitski E, Bonn M, Weckhuysen BM (2009). Angew Chem Int Ed.

[20] Aramburo LR, Karwacki L, Cubillas P, Asahina S, Matthijs de Winter DA, Drury MR, Buurmans ILC, Stavistski E, Mores D, Daturi M, Bazin P, Dumas P, Thibault-Starzyk F, Post JA, Anderson MW, Terasaki O, Weckhuysen BM (2011). Chem Eur J.

[21] Qian Q, Ruiz-Martindez J, Mokhtar M, Asiri AM, Al-Thabaiti SA, Basahel SN, van der Bij HE, Kornatowski J, Weckhuysen BM (2013). Chem Eur J.

[22] Buurmans ILC, Soulimani F, Ruiz-Martinez J, van der Bij HE, Weckhuysen BM (2013). Microporous Mesoporous Mater.

[23] Castro M, Warrender SJ, Wright PA, Apperley DC, Belmabkhout Y, Pirngruber G, Min H-K, Park MB, Hong SB (2009). J Phys Chem C.

[24] Picone AL, Warrender SJ, Slawin AMZ, Dawson DM, Ashbrook SE, Wright PA, Thompson SP, Gaberova L, Llewellyn PL, Moulin B, Vimont A, Daturi M, Park MB, Sung SK, Nam IS, Hong SB (2011). Microporous Mesoporous Mater.

[25] Smith L, Cheetham AK, Marchese L, Thomas JM, Wright PA, Chen JA (1996). Catal Lett.

[26] Martins GAV, Berlier G, Coluccia S, Pastore HO, Superti GB, Gatti G, Marchese L (2007). J Phys Chem C.

[27] Halasz I, Moden B, Petushkov A, Liang JJ, Agarwal M (2015). J Phys Chem C.

[28] Zecchina A, Bordiga S, Spoto G, Scarano D, Spano G, Geobaldo F (1996). J Chem Soc Faraday Trans.

[29] Campbell SM, Jiang X, Howe RF (1999). Microporous Mesoporous Mater.

[30] Evans JC (1960). Spectrochim Acta.

[31] Palmenschikov AG, van Santen AG, Janchen J, Meijer E (1995). J Phys Chem.

[32] Kubelkova L, Novakova J, Nedomova K (1990). J Catal.

[33] Yamazaki H, Shima H, Imai H, Yokoi T, Tatsumi T, Kondo JN (2011). Angew Chem Int Ed.

[34] Jiang Y, Huang J, Reddy Marthala VR, Ooi YS, Weitkamp J, Hunger M (2007). Microporous Mesoporous Mater.

[35] Mores D, Savitski E, Kox MHF, Kornatowski J, Olsbye U, Weckhuysen BM (2008). Chem Eur J.

[36] Howe RF, Taylor MJ (1987). Spectrochim Acta.

[37] Celik FE, Kim T, Mlinar AN, Bell AT (2010). J Catal.

[38] Comes-Vives A, Valla M, Coperet C, Sautet P (2015). ACS Cent Sci.

[39] Liu Y, Muller S, Berger D, Jelic J, Reuter K, Tonigold M, Sanchez-Sanchez M, Lercher J (2016). Angew Chem Int Ed.

